# Novel enaminone derived from thieno [2,3-*b*] thiene: Synthesis, x-ray crystal structure, HOMO, LUMO, NBO analyses and biological activity

**DOI:** 10.1186/s13065-015-0100-9

**Published:** 2015-05-07

**Authors:** Yahia Nasser Mabkhot, Fahad D Aldawsari, Salem S Al-Showiman, Assem Barakat, Saied M Soliman, M Iqbal Choudhary, Sammer Yousuf, Mohammad S Mubarak, Taibi Ben Hadda

**Affiliations:** Department of Chemistry, College of Science, King Saud University, P. O. Box 2455, Riyadh, 11451 Saudi Arabia; King Abdulaziz City for Science and Technology, P. O. Box 6086, Riyadh, 11442 Saudi Arabia; Department of Chemistry, Rabigh College of Science and Art, King Abdulaziz University, P O Box 344, Rabigh, 21911 Saudi Arabia; Department of Chemistry, Faculty of Science, Alexandria University, P.O. Box 426, Alexandria, Ibrahimia 21321 Egypt; H.E.J. Research Institute of Chemistry, International Center for Chemical Sciences, University of Karachi, Karachi, 75270 Pakistan; Department of Chemistry, The University of Jordan, Amman, 11942 Jordan; Laboratoire Chimie Matériaux, FSO, Université Mohammed 1ER, Oujda, 60000 Morocco

**Keywords:** Enaminones, Thieno [2,3-*b*] thiophene, X-ray, HOMO, LUMO

## Abstract

**Background:**

Due to their structural and therapeutic diversity, thienothiophene derivatives have attracted much synthetic interest because of their reactivity and biological activity. The thieno [2,3-*b*] thiophene moiety has been used in the design of a novel pharmaceutical therapies. Additionally, its enaminones derivatives are versatile synthons and have a lot of synthetic applications such as *N*-heterocycles, wide variety of naturally occurring alkaloids and pharmaceutical drugs.

**Results:**

Synthesis of (2*E*,2*′E*)-1,1′-(3,4-diphenylthieno [2,3-*b*] thiophene-2,5-diyl) *bis* (3-(dimethylamino) prop-2-en-1-one) 5 was reported. The structure of compound **5** was deduced by spectroscopic techniques. The compound was crystallizes in the monoclinic system with space group P-1 with cell coordinates a=9.9685 (8) Å, b=10.1382 (8) Å, c=13.3220 (11) Å, *α*=101.018 (2) °, *β*=94.480 (2) °, *γ*=107.207 (1) °, V=1249.3 (1) Å3, and Z=2. In the crystal molecules are packed in chains formed *via* weak intermolecular C21–H21A… O1, C22–H22A…O2 and C27–H27A…O2 hydrogen bondings. Theoretical quantum chemical calculations have been performed on the studied compound using the DFT B3LYP/6-311G (d, p) method. The geometric parameters of the optimized structure are in good agreement with the experimental data obtained from our reported X-ray structure. The two benzene rings and the two side chains are not coplanar with the fused thiophene rings. The electronic spectra of the studied compound have been calculated using the TD-DFT method at the same level of theory. The transition bands at 352.9 nm (f=0.5549) and 332.1 nm (f=0.2190) are due to the H-1 → L (72%) and H → L + 1 (82%) excitations respectively. The NBO calculations were performed to predict the natural atomic charges at the different atomic sites and to study the different intramolecular charge transfer (ICT) interactions occurring in the studied system. It is found that the O and N-atoms have the highest negative charge densities while the S-atoms are the most electropositive. These results give idea about how our molecule could react with the receptor active sites. Compound **5** was evaluated against ant-microbial activity.

**Conclusions:**

Synthesis, molecular structure and spectroscopic invesitgation of (2*E*,2*′E*)-1,1′-(3,4-diphenylthieno [2,3-*b*] thiophene-2,5-diyl) *bis* (3- (dimethylamino) prop-2-en-1-one) **5** was studied.

**Graphical Abstract:**
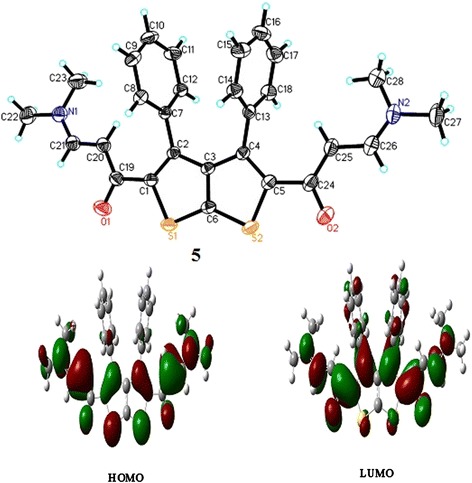
Molecular structure investigation of novel enaminone derived from thieno [2,3-*b*] thiene.

**Electronic supplementary material:**

The online version of this article (doi:10.1186/s13065-015-0100-9) contains supplementary material, which is available to authorized users.

## Background

Enaminones are versatile precursors and have a lot of synthetic applications in organic chemistry. Such compounds are key synthons for the preparation of a wide variety of naturally occurring alkaloids [1,2] and nitrogen-containing heterocycles [3-6]. They have also been employed as important synthesitic intermediate for the synthesis of pharmaceutical drugs with antiviral, and larvicidal [7] anticonvulsant [8-10], properties. Due to their rich applications, many efficient approaches to these compounds have been developed. The structural range and biological importance of functional thienothiophenes has made them attractive research targets over the past decades.

Thienothiophenes skeletons are important in pharmaceutical research because of their versatile biological activities, such as antitumor, antiviral antibacterial, anticancer, antioxidant and α-glucuronidase and α-glucosidase inhibition, antiglaucoma activity, and inhibitors of platelet aggregation properties [11-15]. The thieno [2,3-*b*] thiophenes have been the focus of active research in recent years. For this reason we have focused to prepare certain *bis*-heterocycles containing thieno [2,3-*b*] thiophene derivatives. The molecule 5 that was prepared was found to be potent bacteria and fungus inhibitor.

The skeleton is identified as valuable scaffold for new heterocyclic compounds [16-22]. The structure of (2*E*,2*′E*)-1,1′-(3,4-diphenylthieno [2,3-*b*] thiophene-2,5-diyl) *bis* (3-(dimethylamino) prop-2-en-1-one) (5), was unambiguously deduced by single-crystal X-ray diffraction technique. Also, the DFT/B3LYP calculations have been performed to study the molecular structure characteristics of the studied compound. The natural atomic charges and the intramolecular charge transfer (ICT) interactions were calculated using the NBO method at the same level of theory. The TD-DFT calculations were used to predict the accurate electronic transitions and to draw the HOMO and LUMO levels. Compound **5** was also screened for *in vitro* antimicrobial activity.

## Results and discussion

### Chemistry

**Scheme 1:**
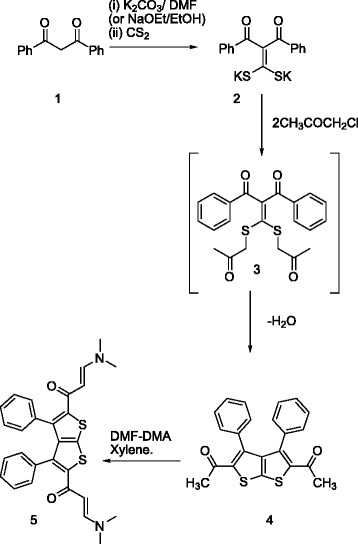
Preparation of the title compound 5.

Compound **5** was synthesized as depicted in Scheme 1, in 75–80% Yield. The structure was deduced by combined use of IR, ^1^H-NMR, ^13^C-NMR, and mass spectral data [23-25]. Accordingly, the assigned structure was unambiguously established *via* single-crystal X-ray diffraction.

### Crystal structure of compound 5

**Figure 1:**
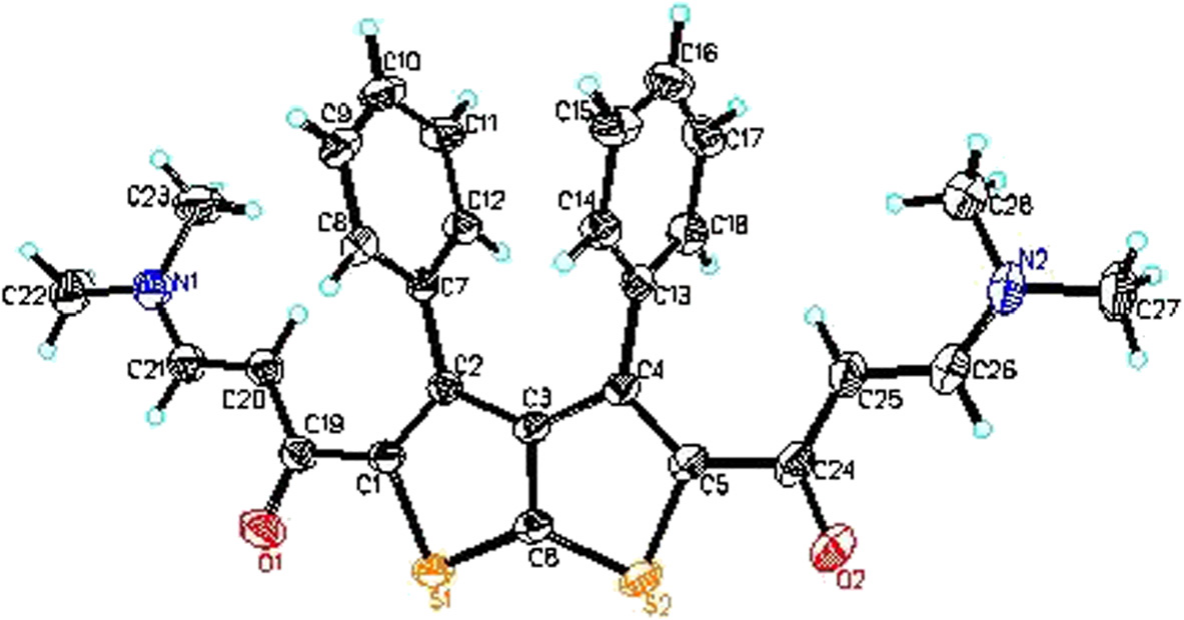
The ORTEP diagram of the final X-ray model of compound 5 with displacement ellipsoids drawn at 30% probability level.

Slow evaporation of glacial acetic acid solution of pure compound **5** yielded colorless crystals. A crystal of dimensions 0.54 × 0.53 × 0.30 mm was selected for X-ray diffraction analysis. Data were collected on a Bruker APEX-II diffractometer equipped with CCD detector and graphite monochromatic Mo Kα radiation (τ=71073 A) at 293 (2) °K. Cell refinement and data reduction were carried out by Bruker SAINT. SHELXS-97 [26,27] was used to solve structure (Table 1). The final refinement was carried out by full-matrix least-squares techniques with anisotropic thermal data for nonhydrogen atoms on *F*2. All the hydrogen atoms were placed in calculated positions. The crystal structure **5** (Figure 1) was finally refined with R factor of 4.43% for 4390 unique reflections. Molecules were found to be packed in crystal lattice through intermolecular hydrogen bonding (Tables 2 and 3).

**Table 1:** **The crystal and experimental data of compound 5**

***Parameters***	
Empirical formula	C_28_ H_26_ N_2_ O_2_ S_2_
Formula weight	486.63
Temperature	100 (2) °K
Wave length	0.71073 Å
Crystal system,	Triclinic,
Space group	P-1
Unit cell dimensions	a=9.9685 (8) Å
	b=10.1382 (8) Å
	c=13.3220 (11) Å
	alpha=101.018 (2)°
	beta=94.480 (2)°
	gamma=107.207 (2)°
Volume	1249.31 (17) A^-3^
Z	2
Calculated density	1.294 Mg/m^-3^
Absorption coefficient	0.241 mm^-1^
F (000)	512
Crystal size	0.37 x 0.34 x 0.15 mm
Theta range for data collection	1.57° to 25.50°.
Limiting indices	-11 <=h <=12,-12 <=k <=11-14 <=l <=16
Reflections collected/unique	7454/4390 [R (int)=0.0182]
Completeness to theta	25.50–94.4%
Absorption correction	Semi-empirical from equivalents
Max. and min. transmission	0.9647 and 0.9161
Refinement method	Full-matrix least-squares on *F* ^-2^
Data/restraints/parameters	4390/0/311
Goodness-of-fit on *F* ^-2^	1.038
Final R indices [I > 2sigma (I)]	R1=0.0443, wR2=0.1124
R indices (all data)	R1=0.0573, wR2=0.1222
Largest diff. peak and hole	0.232 and-0.172 e.A^-3^

**Table 2:** **Selected geometric parameters (Å, °) for 5**

S1—C1	1.750 (2)	C2—C3	1.437 (2)
S1—C6	1.707 (2)	C3—C4	1.440 (3)
S2—C5	1.749 (2)	C4—C5	1.380 (2)
S2—C6	1.714 (2)	C1—C19	1.485 (3)
O1—C19	1.245 (3)	C5—C24	1.494 (3)
O2—C24	1.241 (2)	C19—C20	1.424 (2)
N1—C21	1.333 (2)	C20—C21	1.354 (3)
N2—C26	1.323 (4)	C24—C25	1.412 (4)
C3—C6	1.380 (3)	C25—C26	1.363 (3)
C1—C2	1.378 (3)	C2—C7	1.482 (3)
S1—C6—S2	133.6 (1)	N1—C21—C20	126.7 (2)
S1—C1—C19	114.2 (1)	N2—C26—C25	126.4 (3)
S2—C5—C24	113.8 (1)	C1—C2—C7	125.8 (2)
S1—C1—C2	112.7 (2)	C5—C4—C13	126.7 (2)
S2—C5—C4	113.0 (2)	C2—C3—C4	135.1 (2)
C1—C2—C3	111.1 (2)	C3—C2—C7	123.1 (2)
C5—C4—C3	110.8 (2)	C3—C4—C13	122.5 (2)
S1—C6—C3	113.3 (2)	C2—C1—C19	133.0 (2)
S2—C6—C3	113.1 (2)	C4—C5—C24	133.2 (2)
C6—C3—C2	112.3 (2)	C1—S1—C6	90.6 (1)
C6—C3—C4	112.6 (2)	C5—S2—C6	90.5 (1)
O1—C19—C1	116.4 (2)	C22—N1—C23	117.0 (2)
O2—C24—C5	116.3 (2)	C27—N2—C28	117.5 (2)
S1—C1—C19—O1	8.5 (3)	C25—C26—N2—C28	−2.3 (4)
S2—C5—C24—O2	-8.1 (3)	C20—C21—N1—C23	4.2 (4)
S1—C1—C2—C3	2.0 (2)	C7—C2—C1—C19	8.5 (4)
S1—C6—C3—C2	0.9 (2)	C18—C13—C4—C5	79.4 (3)
S2—C6—C3—C4	1.0 (2)	C13—C4—C5—C24	−1.2 (4)
S2—C5—C4—C3	-1.1 (2)	C7—C2—C3—C4	−4.8 (4)
C1—S1—C6—C3	0.2 (2)	C13—C4—C3—C2	1.3 (4)
C5—S2—C6—C3	-1.4 (2)	O1—C19—C1—C2	−176.5 (2)
O1—C19—C20—C21	5.0 (3)	O2—C24—C25—C26	−1.2 (4)
O2—C24—C25—C26	-1.2 (4)	C25—C24—C5—C4	−10.5 (4)
C19—C20—C21—N1	-178.0 (2)	C20—C19—C1—C2	5.9 (4)

**Table 3:** **Hydrogen bonding data for compound 5**

**D**	**H**	**A**	**D-H**	**H…A**	**D…A**	**D-H…A**
C21	H21A	O1^a^	0.9300	2.5500	3.390 (3)	150.00
C22	H22A	O1^a^	0.9600	2.4000	3.299 (3)	155.00
C27	H27A	O2^b^	0.9600	2.3900	3.232 (3)	146.00

Symmetry codes: ^a^1-x,-y,-z, ^b^2-x,1-y, 2-z.

**Figure 2:**
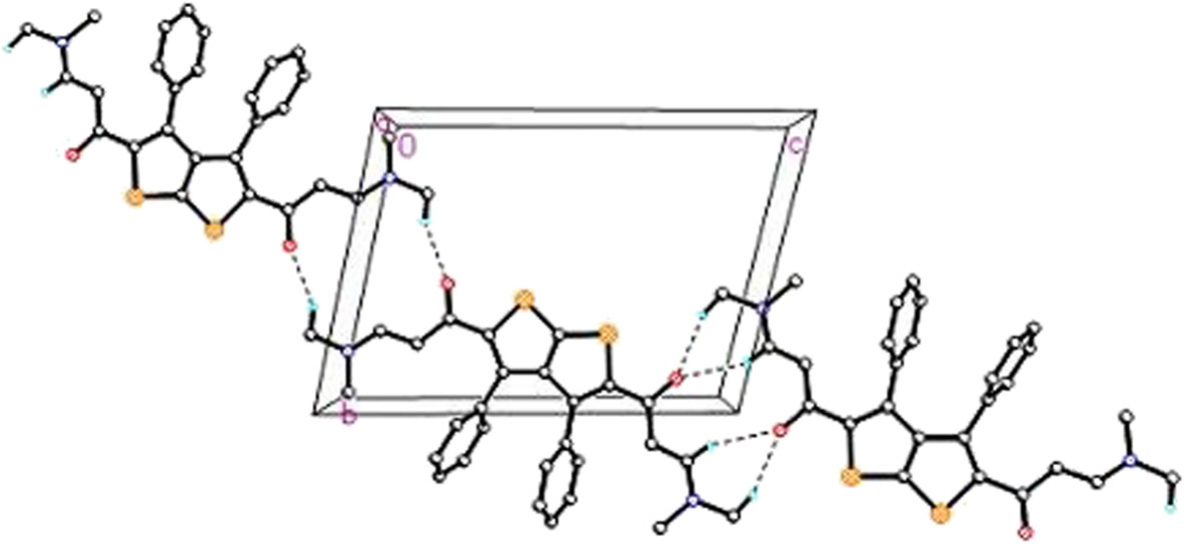
The crystal packing of compound 5. Only the hydrogen atoms involved in bonding can be observed. The rest are omitted for clarity.

The symmetric unit contains two molecules (Z=2). The crystal structure of compound **5** is composed of two planner thiophene rings (S1-C1-C2-C3-C6 and S2-C5-C4-C5-C6) fused along C3 and C6 plane having two phenyl (C7-C12/C13-C18) rings and dimethylaminoprop-2-en-1-one (O1/N1/ C19-C23 and O2/N2/C24-C28) moieties attached to C1and C5 atoms respectively, (Figure 1). Two thiophene (S1/C1-C3/C6 and S2/C5- C3/C6) and phenyl (C7-C12 and C13-C18) rings are each planner with maximum deviation of 0.009 (1) °A for S1 and C5 atoms from the root mean square plane. In the crystal molecules are linked *via* C21–H21A… O1, C22–H22A… O1, C27–H27A… O2 interaction to form chain arranged as observed in Figure 2.

### Computational details

All the quantum chemical calculations of the studied compound were performed by applying DFT method with the B3LYP functional and 6-311G (d, p) basis set using Gaussian 03 software [28]. The input file was taken from the CIF obtained from our reported X-ray single crystal measurement. The geometry was optimized by minimizing the energies with respect to all the geometrical parameters without imposing any molecular symmetry constraints. GaussView4.1 [29] and Chemcraft [30] programs have been used to draw the structures of the optimized geometries. Frequency calculations at the optimized geometry confirmed that the optimized structure is an energy minimum where no imaginary frequency modes were obtained. The electronic spectra of the studied compound were calculated by the TD-DFT method. The natural bond orbital analyses were performed using the NBO calculations as implemented in the Gaussian 03 package [31] at the DFT/B3LYP level.

### Optimized molecular geometry

**Figure 3:**
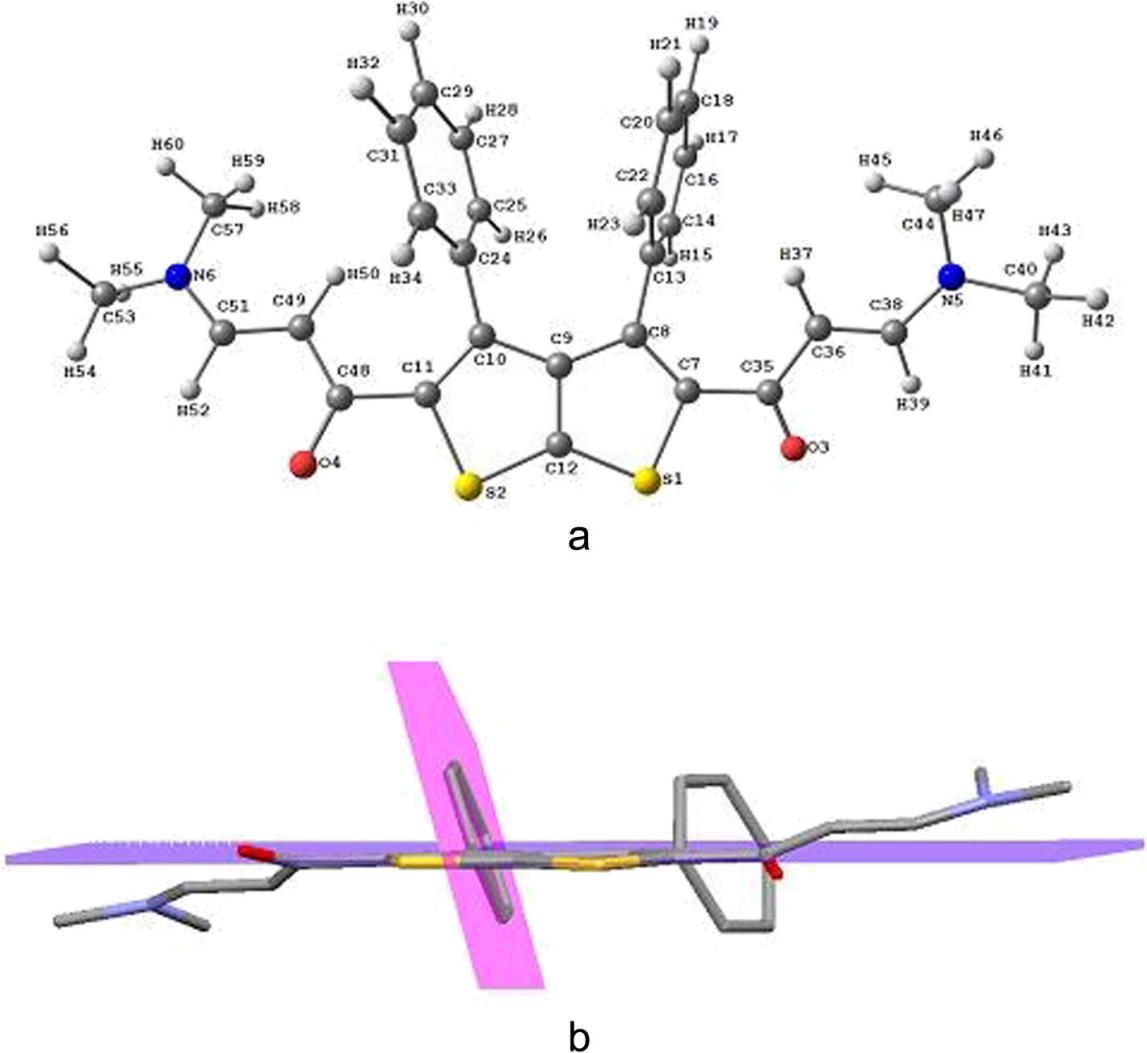
The optimized molecular structure and atom numbering scheme of the studied compound **(a)**; the benzene rings and the side chains are not coplanar with the fused thiophene rings **(b)**.

**Figure 4:**
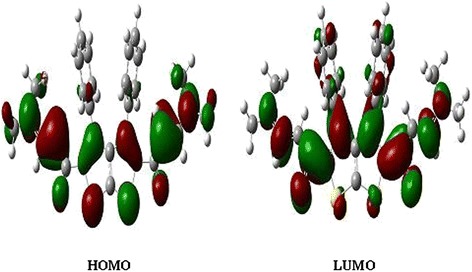
The ground state isodensity surface plots for the frontier molecular orbitals.

The optimized bond lengths and bond angles obtained for the studied compound using the B3LYP method with 6-311G (d, p) basis set are given in Table 4; while the atom numbering of the optimized structure is given in Figure 3. The studied compound possesses C_1_ point group. The optimized geometry of the studied compound is compared with the structural parameters obtained from the CIF data. Generally, the bond lengths and bond angles are predicted very well. Most of bond lengths are overestimated except the C=O bonds. The maximum deviations of the calculated bond length and bond angle values from the experimental data are 0.04 Å (C48–C49) and 4.3° (C48–C49–C51) respectively. These deviations are attributed to the phase difference between the calculations and the experiment. The calculations refer to an isolated molecule in the gas phase, while the experimental data are those for the molecule in the solid phase. Most of the deviations occur for the geometric parameters around the side chain moieties. These side chains have more freedom in the gas phase for rotation around the C-C and C-N bonds causing bigger variations from the solid state molecular structure. The calculated C-C-C bond angle values of the benzene rings are in the range of 118.8–120.6° (exp. 118.4–120.6) [32]. The two benzene rings and the two fused thiophene rings having nearly planar structure where the C-C-C-C and C-C-C-S dihedral angles ring do not exceed 0.47° and 1.16° respectively. The two benzene rings and two side chain moieties are not coplanar with the thiophene ring plane (Figure 4b). The angle between the plane passing through each benzene ring and the fused thiophene ring plane is calculated to be 70.9.

**Table 4:** **The calculated and experimental geometric parameters of the studied compound using DFT B3LYP/6-311G (d, p)**

**Parameter** ^**a**^	**Calc.**	**Exp**	**Parameter**	**Calc.**	**Exp**	**Parameter**	**Calc.**	**Exp**
R (1–7)	1.769	1.750	A (3-35-36)	122.9	124.1	A (14-16-17)	119.7	119.8
R (1–12)	1.721	1.707	A (4-48-11)	117.1	116.3	A (14-16-18)	120.1	120.3
R (2–11)	1.769	1.749	A (4-48-49)	122.9	124.2	A (17-16-18)	120.1	119.9
R (2–12)	1.721	1.714	A (38-5-40)	120.5	121.9	A (16-18-19)	120.2	119.9
R (3–35)	1.235	1.245	A (38-5-44)	120.7	121.0	A (16-18-20)	119.6	120.2
R (4–48)	1.235	1.241	A (5-38-36)	127.8	126.7	A (19-18-20)	120.2	119.9
R (5–38)	1.352	1.333	A (5-38-39)	115.2	116.6	A (18-20-21)	120.1	119.9
R (5–40)	1.455	1.450	A (40-5-44)	117.2	117.0	A (18-20-22)	120.2	120.3
R (5–44)	1.454	1.454	A (5-40-41)	109.5	109.5	A (21-20-22)	119.7	119.9
R (6–51)	1.352	1.323	A (5-40-42)	111.8	109.4	A (20-22-23)	120.0	119.9
R (6–53)	1.455	1.449	A (5-40-43)	110.3	109.4	A (25-24-33)	118.8	118.4
R (6–57)	1.454	1.454	A (5-44-45)	110.2	109.5	A (24-25-26)	119.4	119.7
R (7–8)	1.381	1.378	A (5-44-46)	109.2	109.5	A (24-25-27)	120.6	120.6
R (7–35)	1.491	1.485	A (5-44-47)	111.9	109.4	A (24-33-31)	120.7	120.6
R (8–9)	1.445	1.437	A (51-6-53)	120.5	121.7	A (24-33-34)	119.2	119.7
R (8–13)	1.489	1.482	A (51-6-57)	120.7	120.8	A (26-25-27)	120.0	119.7
R (9–10)	1.445	1.440	A (6-51-49)	127.8	126.4	A (25-27-28)	119.7	119.8
R (9–12)	1.395	1.380	A (6-51-52)	115.2	116.7	A (25-27-29)	120.2	120.3
R (10–11)	1.381	1.380	A (53-6-57)	117.2	117.5	A (28-27-29)	120.1	119.9
R (10–24)	1.489	1.485	A (6-53-54)	109.5	109.5	A (27-29-30)	120.2	120.0
R (11–48)	1.491	1.494	A (6-53-55)	111.8	109.5	A (27-29-31)	119.6	120.0
R (13–14)	1.400	1.382	A (6-53-56)	110.3	109.5	A (30-29-31)	120.2	120.0
R (13–22)	1.400	1.384	A (6-57-58)	111.9	109.5	A (29-31-32)	120.1	120.0
R (14–16)	1.393	1.382	A (6-57-59)	110.2	109.4	A (29-31-33)	120.1	120.2
R (16–18)	1.394	1.365	A (6-57-60)	109.2	109.5	A (32-31-33)	119.7	119.9
R (18–20)	1.394	1.367	A (8-7-35)	134.9	133.0	A (31-33-34)	120.2	119.7
R (20–22)	1.392	1.379	A (7-8-9)	111.7	111.1	A (35-36-37)	119.7	119.1
R (24–25)	1.400	1.376	A (7-8-13)	124.1	125.8	A (35-36-38)	118.2	121.8
R (24–33)	1.400	1.390	A (7-35-36)	120.0	119.5	A (37-36-38)	122.1	119.1
R (25–27)	1.392	1.381	A (9-8-13)	124.1	123.1	A (36-38-39)	117.0	116.7
R (27–29)	1.394	1.370	A (8-9-10)	137.0	135.1	A (41-40-42)	108.3	109.6
R (29–31)	1.394	1.360	A (8-9-12)	111.5	112.3	A (41-40-43)	108.5	109.5
R (31–33)	1.393	1.380	A (8-13-14)	120.2	119.8	A (42-40-43)	108.3	109.4
R (35–36)	1.456	1.424	A (8-13-22)	121.0	121.0	A (45-44-46)	108.5	109.5
R (36–38)	1.362	1.354	A (10-9-12)	111.5	112.6	A (45-44-47)	108.4	109.5
R (48–49)	1.456	1.412	A (9-10-11)	111.7	110.8	A (46-44-47)	108.7	109.5
R (49–51)	1.362	1.363	A (9-10-24)	124.1	122.5	A (48-49-50)	119.7	118.8
A (7-1-12)	90.0	90.6	A (11-10-24)	124.1	126.7	A (48-49-51)	118.2	122.4
A (1-7-8)	112.8	112.7	A (10-11-48)	134.9	133.2	A (50-49-51)	122.1	118.8
A (1-7-35)	112.3	114.2	A (10-24-25)	121.0	121.1	A (49-51-52)	117.0	116.8
A (1-12-2)	132.1	133.6	A (10-24-33)	120.2	120.5	A (54-53-55)	108.3	109.4
A (1-12-9)	113.9	113.3	A (14-13-22)	118.8	119.1	A (54-53-56)	108.5	109.4
A (11-2-12)	90.0	90.5	A (13-14-15)	119.2	120.0	A (55-53-56)	108.3	109.4
A (2-11-10)	112.8	113.0	A (13-14-16)	120.7	120.0	A (58-57-59)	108.4	109.5
A (2-11-48)	112.3	113.8	A (13-22-20)	120.6	120.1	A (58-57-60)	108.7	109.5
A (2-12-9)	113.9	113.1	A (13-22-23)	119.4	120.0	A (59-57-60)	108.5	109.5
A (3-35-7)	117.1	116.4	A (15-14-16)	120.2	120.0			

^a^The calculated dihedral angles given in Additional file 1: Table S1.

In agreement with the X-ray structure, the DFT calculations predicted the S---O intramolecular distances to be 2.794 Å (exp. 2.799–2.804 Å). This value is smaller than the sum of the Batsanov’s van der Waal radii of the two elements [33] which indicate the presence of some intramolecular S---O interactions. These intramolecular noncovalent interactions play crucial role in modulating the conformational preferences of the molecule as well as in applications of drug design [34].

### Natural atomic charge

Distribution of positive and negative charges has vital role in the application of quantum chemical calculations to molecular system because of atomic charges affect dipole moment, molecular polarizability, electronic structure, acidity–basicity behavior and more lot of properties of molecular system [35]. These electronic properties have strong relations to the biological activity of compound. In this regards, the natural atomic charges (NAC) were calculated using the DFT B3LYP/6-311G (d, p) and the results were given in Table 5. The studied molecule has O, N and S heteroatoms able to interact with the receptor reactive sites. The O and N-atoms have negative natural charge densities where the O-atoms are the most electronegative atomic sites in the molecule. The natural atomic charges at the O and N-atoms are calculated to be-0.6275 and-0.4109 respectively. In contrast, the S-atoms have electropositive nature with NAC value around +0.4768. The negatively charged atomic sites (O and N) are more likely to interact with positive part of the receptor. On the contrary, the most positively charged part (S-atom) will interact quite easily with negatively charged part of the receptor. These interactions can play crucial role in bioactivity of the studied compound.

**Table 5:** **The natural atomic charges calculated at the B3LYP/6-311G (d, p)**

**Atom**	**NAC**	**Atom**	**NAC**
S1	0.4769	C31	-0.1992
S2	0.4768	H32	0.2015
O3	-0.6275	C33	-0.1815
O4	-0.6275	H34	0.2109
N 5	-0.4109	C35	0.4817
N6	-0.4109	C36	-0.4302
C7	-0.2120	H37	0.2135
C8	-0.0515	C38	0.1238
C9	-0.1166	H39	0.2097
C10	-0.0515	C40	-0.3385
C11	-0.2120	H41	0.2007
C12	-0.3588	H42	0.1793
C13	-0.0517	H43	0.1853
C14	-0.1815	C44	-0.3611
H15	0.2109	H45	0.2070
C16	-0.1992	H46	0.1917
H17	0.2015	H47	0.1857
C18	-0.2043	C48	0.4817
H19	0.1998	C49	-0.4302
C20	-0.1920	H50	0.2135
H21	0.2019	C51	0.1238
C22	-0.1817	H52	0.2097
H23	0.2106	C53	-0.3385
C24	-0.0517	H54	0.2007
C25	-0.1817	H55	0.1793
H26	0.2106	H56	0.1853
C27	-0.1920	C57	-0.3611
H28	0.2019	H58	0.1857
C29	-0.2044	H59	0.2070
H30	0.1998	H60	0.1917

Moreover, all the C-atoms have moderate negative natural charge values except C35, C38, C48 and C51 atoms which are electropositive. The most electropositive C-sites are C48 and C51 as these atoms bonded to the highest electronegative atoms (O) in the molecule. All the H-atoms are electropositive with NAC values in the range of 0.1793–0.2135.

### Frontier molecular orbitals

**Figure 5:**
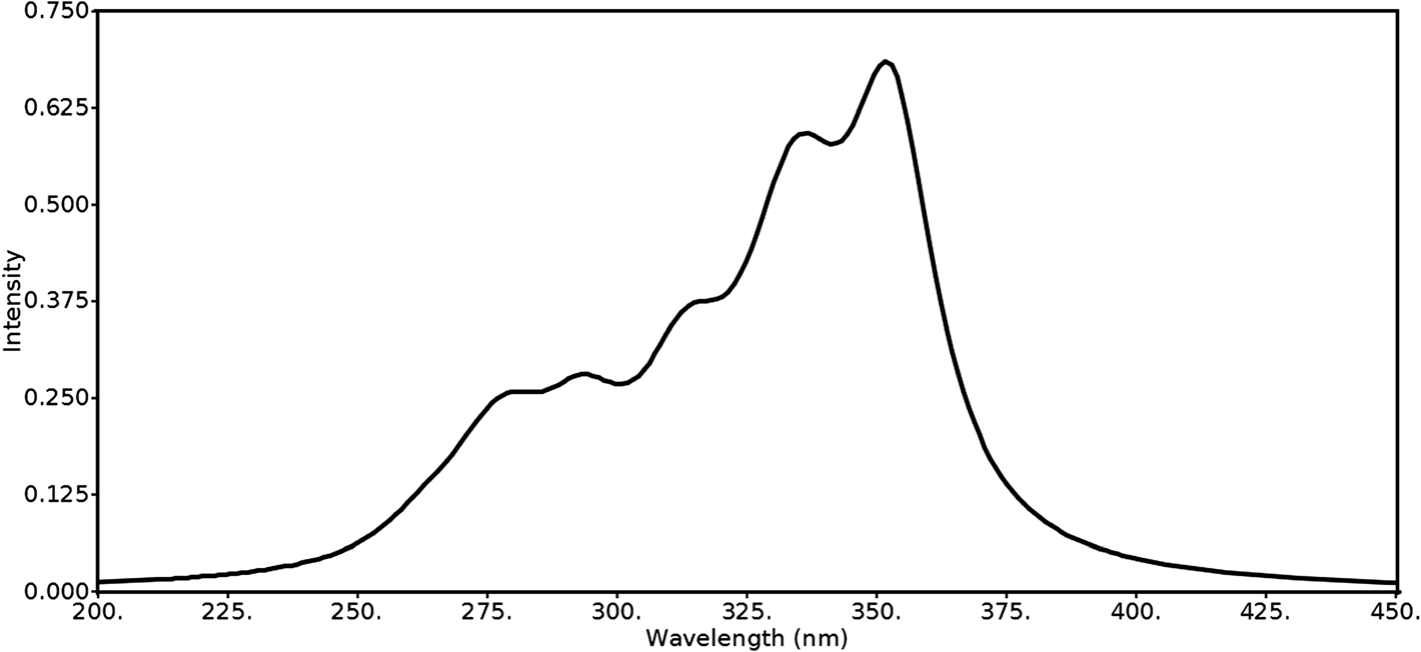
The calculated electronic spectra of the studied compound using TD-DFT method.

The energies and electron densities of the frontier molecular orbitals, HOMO and LUMO, are important electronic parameters. The latter were used to determine the most reactive sites in the unsaturated system [36]. Also, the HOMO-LUMO energy difference (ΔE) is a measure of the intramolecular charge transfer and was used in biological activity studies [37,38]. The HOMO and LUMO pictures calculated using the B3LYP/6-311G (d, p) are shown in Figure 4. The E_HOMO_ and E_LUMO_ values are-5.5811 and-1.5962 eV respectively. The ΔE value represents the smallest amount of energy needed for electronic excitation which belongs mainly to π-π* transition. In the studied compound, the HOMO-LUMO energy gap (ΔE) of the studied compound is 3.9849 eV. The more accurate electronic transitions have been calculated using the time-dependant density functional theory (TD-DFT). The calculated electronic spectra using the TD-DFT method is shown in Figure 5. The results of the TD calculations which represent the calculated λ_max_ values with their major contributions of molecular orbitals to the formation of bands are given in Additional file 1: Table S2. The electronic transition bands calculated at 352.9 nm (f = 0.5549) and 332.1 nm (f = 0.2190) are due to the H-1 → L (72%) and H → L + 1 (82%) excitations respectively. The other electronic transitions which occur at lower λ_max_ values of 314.0 nm (f = 0.1076), 293.4 nm (f = 0.1365) and 278.9 nm (f = 0.0541) are predicted to have lower absorption intensities.

### Nonlinear optical properties

Nonlinear optical materials were used as key materials for photonic communications which use light instead of electron for data transmission. With the development of laser technology, nonlinear optical materials have been extensively applied to industry, national defense, medicine and research [39,40]. Several organic materials were used for such applications. These organic compounds were characterized by their high polarizability (α_0_) and low HOMO-LUMO gap (ΔE). The α_0_ value of the studied compound is calculated to be 409.93 Bohr^3^ which is 15 times higher than the urea, the reference used for comparison of the NLO activity [32]. Moreover, the small HOMO-LUMO energy gap is an important requirement for NLO materials. The small ΔE value indicates low transition energy and hence more shift for electronic transition band to the visible region. The studied compound has lower (3.9849 eV) energy gap (ΔE) than urea (7.6644 eV). Based on these calculations, the studied compound could be predicted as better NLO material than urea [41].

### Natural bond orbital analysis

The stabilization energies E ^(2)^ due to intramolecular charge transfer (ICT) interactions have been calculated using the second-order perturbation theory [42]. The significant ICT interactions are given in Table 6. The E ^(2)^ values indicate the intensity of the electron delocalization *i.e.* extent of conjugation of the whole system [43]. The ICT interactions formed by the orbital overlap between π → π*, n → π* and n → σ* causing stabilization of the system upto 51.63 kcal/mol. The most significant interactions in this molecule, is electron donation from LP (1) N to the antibonding π*–NBO of the adjacent C=C bonds. For example, the ICT interactions from the LP (1) N5/ LP (1) N6 to the antibonding π*(C36–C38)/C49–C51 having E ^(2)^ of 51.63 kcal/mol. Also, the π (C36–C38) → π*(O3–C35) and π (C49–C51) → π*(O4–C48) interactions resulting in stabilization of the system by 26.57 kcal/mol. These results indicate the presence of strong electron delocalizations between the C=C, C=O and the lone pair of the N-atom of the side chain moiety.

**Table 6:** **The second order perturbation energies E** ^**(2)**^ **(kcal/mol) of the most important charge transfer interactions in the studied compound using B3LYP method**

**Donor NBO (i)**	**Acceptor NBO (j)**	**E** ^**(2)**^ **kcal/mol**
BD (1) S1-C7	BD*(1) S2-C12	7.19
BD (1) S2-C11	BD*(1) S1-C12	7.19
BD (2) C7-C8	BD*(2) O3-C35	16.53
BD (2) C7-C8	BD*(2) C9-C12	13.57
BD (2) C9-C12	BD*(2) C7-C8	15.53
BD (2) C9-C12	BD*(2) C10-C11	15.53
BD (2) C10-C11	BD*(2) O4-C48	16.53
BD (2) C10-C11	BD*(2) C9-C12	13.57
BD (2) C13-C14	BD*(2) C16-C18	21.16
BD (2) C13-C14	BD*(2) C20-C22	20.01
BD (2) C16-C18	BD*(2) C13-C14	20.29
BD (2) C16-C18	BD*(2) C20-C22	20.10
BD (2) C20-C22	BD*(2) C13-C14	20.88
BD (2) C20-C22	BD*(2) C16-C18	20.86
BD (2) C24-C33	BD*(2) C25-C27	20.01
BD (2) C24-C33	BD*(2) C29-C31	21.16
BD (2) C25-C27	BD*(2) C24-C33	20.88
BD (2) C25-C27	BD*(2) C29-C31	20.86
BD (2) C29-C31	BD*(2) C24-C33	20.29
BD (2) C29-C31	BD*(2) C25-C27	20.10
BD (2) C36-C38	BD*(2) O3-C35	26.57
BD (2) C49-C51	BD*(2) O4-C48	26.57
LP (2) S1	BD*(2) C7-C8	19.64
LP (2) S1	BD*(2) C9-C12	24.25
LP (2) S1	BD*(2) C10-C11	19.64
LP (2) O3	BD*(1) C7-C35	17.56
LP (2) O3	BD*(1) C35-C36	17.01
LP (2) O4	BD*(1) C11-C48	17.56
LP (2) O4	BD*(1) C48-C49	17.01
LP (1) N5	BD*(2) C36-C38	51.63
LP (1) N6	BD*(2) C49-C51	51.63

Interestingly, the studied compound has electron deficient bivalent sulphur atoms (~ + 0.4769) have relatively low-lying σ*-orbitals (0.209-0.235 A.U.) related to the C-S bonds that are available for interaction with the electron donor O-atoms neighboring to them. The NBO calculations predicted the energies of the σ*(C-S) NBO are 0.209–0.235 A.U. the ICT interaction energies due to the LP (2) O → σ*(S1–C12) LP (2) O4 → σ*(S2–C12) are calculated to be 1.32 Kcal/mol indicating the weak S---O interactions. The occupancies of the σ*(S1–C7) and σ*(S1–C12) are 0.0279 and 0.0325 due to the loss of the occupancies from the localized NBO of the Lewis structure into these empty non-Lewis NBOs.

### Antimicrobial activity of compound 5

Compound **5** was tested against its antimicrobial activity representing Gram-negative bacteria (*Escherichia coli* and *Pseudomonas aeruginosa*), Gram-positive bacteria (*Staphylococcus pneumoniae* and *Bacillus subtilis*), and fungi (*Candida albicans* and *Aspergillus fumigatus*), and the activities were compared with standard antimicrobial drug, specified in US pharmacopeia at 25 μg/mL. Compound **5** showed a relatively moderate inhibitory effect against Gram positive bacteria (*Bacillus subtilis* and *Staphylococcus pneumoniae*) as compared to standard drug ampicillin (Table 7).

**Table 7:** **Antimicrobial activity of compound 5 (Zone of inhibition; diameter in mm**

**Compd.**	**Fungi**		**Gram (+) bacteria**		**Gram (−) bacteria**	
	**(A)**	**(B)**	**(C)**	**(D)**	**(E)**	**(F)**
**SD**	23.7 ± 0.1	25.4 ± 0.1	23.8 ± 0.2	32.4 ± 0.3	17.3 ± 0.1	19.9 ± 0.3
**5**	17.3 ± 0.44	16.9 ± 0.25	16.3 ± 0.55	18.3 ± 0.25	NA	NA

ST=25 μg/mL. (A): As*pergillus fumigatus*; (B): *Saccharomyces cerevisiae*; (C): Staphylococcus aureus; (D): *Bacilils subtilis*; (E): *Pseudomonas aeruginosa*; (F): *Escherichia coli*. NA: Not Active. The test was performed three times for each bacterium. Streptomycin and Clotrimazole were used as antibacterial and antifungal standard drugs respectively.

### Experimental section

#### General

##### General

All the chemicals were purchased from various suppliers, including Sigma-Aldrich and Fluka, and were used without further purification, unless otherwise stated. All melting points were measured on a Gallenkamp melting point apparatus in open glass capillaries and are uncorrected. IR Spectrum was recorded as KBr pellets on a Nicolet 6700 FT-IR spectrophotometer. The NMR spectra were recorded on a Varian Mercury Jeol-400 NMR spectrometer. ^1^HNMR (400 MHz) and ^13^C-NMR (100 MHz) were run in deuterated dimethyl sulphoxide (DMSO-*d*_6_). Chemical shifts (δ) are referred to in ppm while *J*-coupling constants were represented in *Hz*. Mass spectra were recorded on a Jeol of JMS-600 H. Elemental analysis was carried out on Elmer 2400 Elemental Analyzer, CHN mode. The single-crystal X-ray diffraction measurements were performed using Bruker SMART APEX II CCD diffractometer.

### Preparation of (2E,2′E)-1,1′-(3,4-diphenylthieno [2,3-b] thiophene-2,5-diyl) bis (3-(dimethylamino) prop-2-en-1-one) 5

A mixture of compound 1,1′-(3,4-diphenylthieno [2,3-*b*] thiophene-2,5-diyl) diethanone **4** [37] (1.89 g, 5 mmol), DMF-DMA (1.19 mL, 0.01 mol) was refluxed in *m*-xylene (15 mL) for 7 h. After cooling, the resulting solid product was collected by filtration and recrystallization by using DMF/EtOH to give the desired product **1**. Yield: 73%; m.p. 250°C; IR (_νmax_): 1622 (C=O), 1539, 1457 cm^-1^; ^1^H-NMR (400Hz, DMSO-*d*_6_) δ (ppm): 2.99 (s, 12H, CH_3_), 4.53 (d, 1H, *J*=12 Hz, CH), 5.38 (d, 1H, *J*=12 Hz, CH), 7.41–7.65 (m, 5H, C_6_H_5_); ^13^C-NMR (100Hz, DMSO-*d*_6_) δ (ppm): 192.2, 153.2, 147.7, 145.8, 141.8, 138.8, 134.8, 129.8, 129.6, 129.2, 125.3, 30.6; MS *m/z* (%): 488 [M+, 30%], 386 (100), 368 (47), 213 (73), 43 (46); Anal. calcd. for C_28_H_26_N_2_O_2_S_2_: C, 69.11; H, 5.39; N, 5.76; Found: C, 69.15; H, 5.41; N, 5.78.

### Antifungal activity of compound (5)

Tested sample was screened *in vitro* for its antifungal activity against various fungi, namely, *Aspergillus fumigatus* (RCMB 002568) and *Candida albicans* (RCMB 05036). The antifungal activity was performed by agar well diffusion method.

Fungal strains were grown in 5 mL sabouraud dextrose broth (glucose/peptone; 40/10) for 3–4 days to obtain 105 CFU/mL cells. The fungal culture (0.1 mL) was spread uniformly on the sabouraud dextrose agar plates by sterilized triangular folded glass rod. Plates were left for 5–10 min so the culture is properly adsorbed on the surface. Now small wells of size 4 mm × 2 mm were cut into the plates with the help of well cutter and bottom of the wells was sealed with 0.8% soft agar to prevent the flow of test sample at the bottom of the well. 100 μL of the tested samples (10 mg/mL) was loaded into the wells of the plates. Compound **5** dissolved in DMSO, while pure DMSO was also used as control. The plates were kept for incubation at 30°C for 3–4 days and then examined for the formation of zones of inhibition. The test was performed three times for each fungus. Amphotericin B was used as standard antifungal drug.

### Antibacterial activity of compound (5)

Antibacterial activities were investigated by using agar well diffusion method, against the *Staphylococcus pneumonia* (RCMB 010010) and *Bacillus subtilis* (RCMB 010067) {as Gram-positive bacteria} and *Pseudomonas aeruginosa* (RCMB 010043) and *Escherichia coli* (RCMB 0100052) {as Gram-negative bacteria}. The solution of 5 mg/mL of compound in DMSO was prepared for testing against bacteria. Centrifuged pellets of bacteria from 24 h old culture containing approximately 104–106 CFU (colony forming unit) per mL were spread on the surface of nutrient agar (type tone 1%, yeast extract 0.5%, NaCl 0.5%, agar, and 1000 mL of distilled water, pH 7.0) which was autoclaved under 121°C for at least 20 min. Wellswere created in medium with the help of sterile metallic bores and then cooled down to 45°C. The activity was determined by measuring the diameter of the inhibition zone (in mm). A volume of 100 μL of the tested samples (10 mg/mL) was loaded into the wells of the plates. Solution of compound was prepared in DMSO while DMSO was also loaded as control. The plates were kept for incubation at 37°C for 24 h and then the plates were examined for the formation of zone of inhibition. Each inhibition zone was measured three times by caliper to get an average value. The test was performed three times for each bacterium. Ampicillin and was used as antibacterial standard drug.

## Conclusion

The synthesis and characterization of a new 1,1′-(3,4-diphenylthieno [2,3-*b*] thiene-2,5-diyl) bis (3-dimethylaminoprop-2-en-1-one) (**5**) were successfully achieved in high yield. The DFT B3LYP/6-311G (d, p) method were used to calculate the optimized molecular structure of the studied compound. The optimized molecular structure showed good agreement with our reported X-ray crystal structure. The natural atomic charges were calculated using the NBO method. The O and S atoms are the most probable sites to react with the active sites of the receptor molecule. The α_0_ and HOMO-LUMO energy gap (ΔE) values indicated that the studied molecule is considered as a better NLO material than urea. The calculated electronic spectra using TD-DFT method showed two intense transition bands at 352.9 nm (f = 0.5549) and 332.1 nm (f = 0.2190) due to the H-1 → L (72%) and H → L + 1 (82%) transitions respectively. There is strong electron delocalization from the Lp (1) N to the adjacent C=C and extended to the C=O group.

## Additional file

Additional file 1:
**Additional information.**
