# Supplementary material for: Novel enaminone derived from thieno [2,3-b] thiene: Synthesis, x-ray crystal structure, HOMO, LUMO, NBO analyses and biological activity
Source: Chem Cent J. 2015 May 7;9:24. doi: 10.1186/s13065-015-0100-9 (PMC4461800; doi:10.1186/s13065-015-0100-9)
Supplement: Additional file 1: — Additional information. [file 13065_2015_100_MOESM1_ESM.doc]

**Table S1** The calculated dihedral angles of the studied compound.

| **Parameter** | **Calc.** | **Parameter** | **Calc.** | **Parameter** | **Calc.** |
| --- | --- | --- | --- | --- | --- |
| D(12,1,7,8) | -1.3 | D(8,7,35,36) | 15.1 | D(19,18,20,21) | 0.0 |
| D(12,1,7,35) | 177.9 | D(7,8,9,10) | -179.9 | D(19,18,20,22) | 179.7 |
| D(7,1,12,2) | -178.6 | D(7,8,9,12) | 0.1 | D(18,20,22,13) | 0.4 |
| D(7,1,12,9) | 1.4 | D(13,8,9,10) | -2.3 | D(18,20,22,23) | 179.9 |
| D(12,2,11,10) | -1.3 | D(13,8,9,12) | 177.7 | D(21,20,22,13) | -180.0 |
| D(12,2,11,48) | 177.9 | D(7,8,13,14) | 69.7 | D(21,20,22,23) | -0.4 |
| D(11,2,12,1) | -178.6 | D(7,8,13,22) | -110.1 | D(10,24,25,26) | -0.3 |
| D(11,2,12,9) | 1.4 | D(9,8,13,14) | -107.6 | D(10,24,25,27) | 179.3 |
| D(40,5,38,36) | 168.4 | D(9,8,13,22) | 72.7 | D(33,24,25,26) | 180.0 |
| D(40,5,38,39) | -12.5 | D(8,9,10,11) | -179.9 | D(33,24,25,27) | -0.5 |
| D(44,5,38,36) | 3.2 | D(8,9,10,24) | -2.3 | D(10,24,33,31) | -179.5 |
| D(44,5,38,39) | -177.7 | D(12,9,10,11) | 0.1 | D(10,24,33,34) | 0.2 |
| D(38,5,40,41) | 18.7 | D(12,9,10,24) | 177.7 | D(25,24,33,31) | 0.2 |
| D(38,5,40,42) | -101.4 | D(8,9,12,1) | -1.2 | D(25,24,33,34) | 179.9 |
| D(38,5,40,43) | 138.1 | D(8,9,12,2) | 178.8 | D(24,25,27,28) | -180.0 |
| D(44,5,40,41) | -175.6 | D(10,9,12,1) | 178.8 | D(24,25,27,29) | 0.4 |
| D(44,5,40,42) | 64.3 | D(10,9,12,2) | -1.2 | D(26,25,27,28) | -0.4 |
| D(44,5,40,43) | -56.2 | D(9,10,11,2) | 0.9 | D(26,25,27,29) | 179.9 |
| D(38,5,44,45) | -46.1 | D(9,10,11,48) | -178.0 | D(25,27,29,30) | 179.7 |
| D(38,5,44,46) | -165.2 | D(24,10,11,2) | -176.6 | D(25,27,29,31) | 0.0 |
| D(38,5,44,47) | 74.5 | D(24,10,11,48) | 4.4 | D(28,27,29,30) | 0.0 |
| D(40,5,44,45) | 148.2 | D(9,10,24,25) | 72.7 | D(28,27,29,31) | -179.7 |
| D(40,5,44,46) | 29.1 | D(9,10,24,33) | -107.6 | D(27,29,31,32) | 179.8 |
| D(40,5,44,47) | -91.2 | D(11,10,24,25) | -110.1 | D(27,29,31,33) | -0.2 |
| D(53,6,51,49) | 168.4 | D(11,10,24,33) | 69.7 | D(30,29,31,32) | 0.1 |
| D(53,6,51,52) | -12.5 | D(2,11,48,4) | 13.3 | D(30,29,31,33) | -179.9 |
| D(57,6,51,49) | 3.2 | D(2,11,48,49) | -163.9 | D(29,31,33,24) | 0.1 |
| D(57,6,51,52) | -177.7 | D(10,11,48,4) | -167.7 | D(29,31,33,34) | -179.6 |
| D(51,6,53,54) | 18.7 | D(10,11,48,49) | 15.1 | D(32,31,33,24) | -179.9 |
| D(51,6,53,55) | -101.4 | D(8,13,14,15) | 0.2 | D(32,31,33,34) | 0.4 |
| D(51,6,53,56) | 138.1 | D(8,13,14,16) | -179.5 | D(3,35,36,37) | -176.3 |
| D(57,6,53,54) | -175.6 | D(22,13,14,15) | 179.9 | D(3,35,36,38) | 2.0 |
| D(57,6,53,55) | 64.3 | D(22,13,14,16) | 0.2 | D(7,35,36,37) | 0.8 |
| D(57,6,53,56) | -56.2 | D(8,13,22,20) | 179.3 | D(7,35,36,38) | 179.0 |
| D(51,6,57,58) | 74.5 | D(8,13,22,23) | -0.3 | D(35,36,38,5) | 178.8 |
| D(51,6,57,59) | -46.1 | D(14,13,22,20) | -0.5 | D(35,36,38,39) | -0.3 |
| D(51,6,57,60) | -165.2 | D(14,13,22,23) | 180.0 | D(37,36,38,5) | -3.1 |
| D(53,6,57,58) | -91.2 | D(13,14,16,17) | -179.9 | D(37,36,38,39) | 177.9 |
| D(53,6,57,59) | 148.2 | D(13,14,16,18) | 0.1 | D(4,48,49,50) | -176.3 |
| D(53,6,57,60) | 29.1 | D(15,14,16,17) | 0.4 | D(4,48,49,51) | 2.0 |
| D(1,7,8,9) | 0.9 | D(15,14,16,18) | -179.6 | D(11,48,49,50) | 0.8 |
| D(1,7,8,13) | -176.6 | D(14,16,18,19) | -179.9 | D(11,48,49,51) | 179.0 |
| D(35,7,8,9) | -178.0 | D(14,16,18,20) | -0.2 | D(48,49,51,6) | 178.8 |
| D(35,7,8,13) | 4.4 | D(17,16,18,19) | 0.1 | D(48,49,51,52) | -0.3 |
| D(1,7,35,3) | 13.3 | D(17,16,18,20) | 179.8 | D(50,49,51,6) | -3.1 |
| D(1,7,35,36) | -163.8 | D(16,18,20,21) | -179.7 | D(50,49,51,52) | 177.9 |
| D(8,7,35,3) | -167.7 | D(16,18,20,22) | 0.0 |  |  |

Table S2 The calculated electronic transition bands using TD-DFT method.

| Wavelength (nm) | f | Major contributions |
| --- | --- | --- |
| 352.9 | 0.5549 | H-1→L (72%) |
| 347.5 | 0.0006 | H→L (85%) |
| 338.1 | 0.0030 | H-5→L (50%), H-4→L+1 (28%) |
| 336.9 | 0.1899 | H-5→L+1 (21%), H-4→L (43%), H-1→L (15%) |
| 332.1 | 0.2190 | H→L+1 (82%) |
| 322.8 | 0.0109 | H-1→L+1 (84%) |
| 314.0 | 0.1076 | H-3→L+1 (17%), H-2→L (58%) |
| 312.5 | 0.0855 | H-3→L (80%) |
| 302.1 | 0.0036 | H-2→L+1 (84%) |
| 293.4 | 0.1365 | H-3→L+1 (68%), H-2→L (13%) |
| 280.3 | 0.0130 | H-1→L+4 (11%), H→L+2 (78%) |
| 279.8 | 0.0359 | H-6→L (27%), H-1→L+2 (42%), H→L+4 (16%) |
| 278.9 | 0.0541 | H-6→L (35%), H-4→L (10%), H-1→L+2 (27%) |
| 275.0 | 0.0372 | H-1→L+5 (10%), H→L+3 (78%) |
| 273.7 | 0.0000 | H-5→L (17%), H-4→L+1 (34%), H-1→L+3 (37%) |
| 272.9 | 0.0235 | H-5→L (14%), H-4→L+1 (29%), H-1→L+3 (41%) |
| 269.9 | 0.0007 | H-6→L (29%), H-5→L+1 (52%), H-4→L (15%) |
| 267.3 | 0.0036 | H-2→L+2 (11%), H-1→L+2 (19%), H→L+4 (64%) |
| 265.6 | 0.0007 | H-3→L+2 (11%), H-1→L+4 (77%) |
| 262.8 | 0.0349 | H-6→L+1 (71%) |
